# Supplementary material for: Genetic architecture of plasma metabolome in 254,825 individuals
Source: Nat Commun. 2025 Sep 19;16:8272. doi: 10.1038/s41467-025-62126-w (PMC12449471; doi:10.1038/s41467-025-62126-w)
Supplement: Supplementary file 1 — Supplementary Information [file 41467_2025_62126_MOESM1_ESM.pdf]

Supplementary Figures

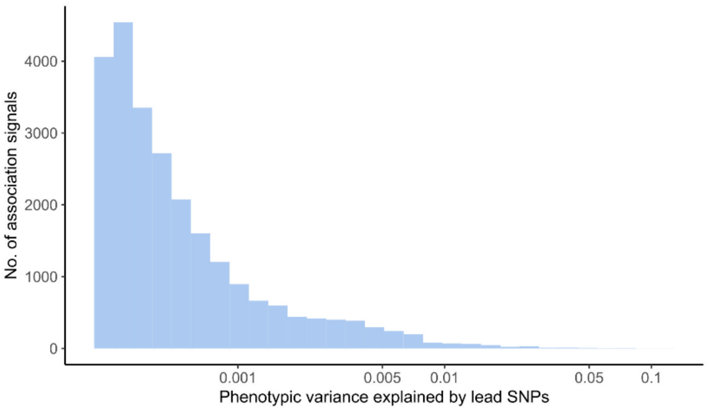

Supplementary Figure 1. Distribution of metabolite phenotypic variance explained by lead variants.

The distribution is plotted on the log scale.

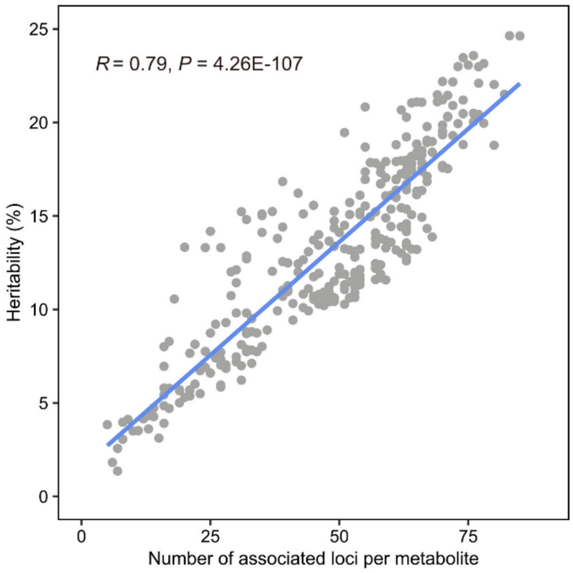

Supplementary Figure 2. Scatterplot illustrating the relationship between the heritability of metabolites and the number of associated genetic loci.

The fitted line was derived from a linear regression, with corresponding Spearman's correlation coefficient and two-sided *P*-value provided.

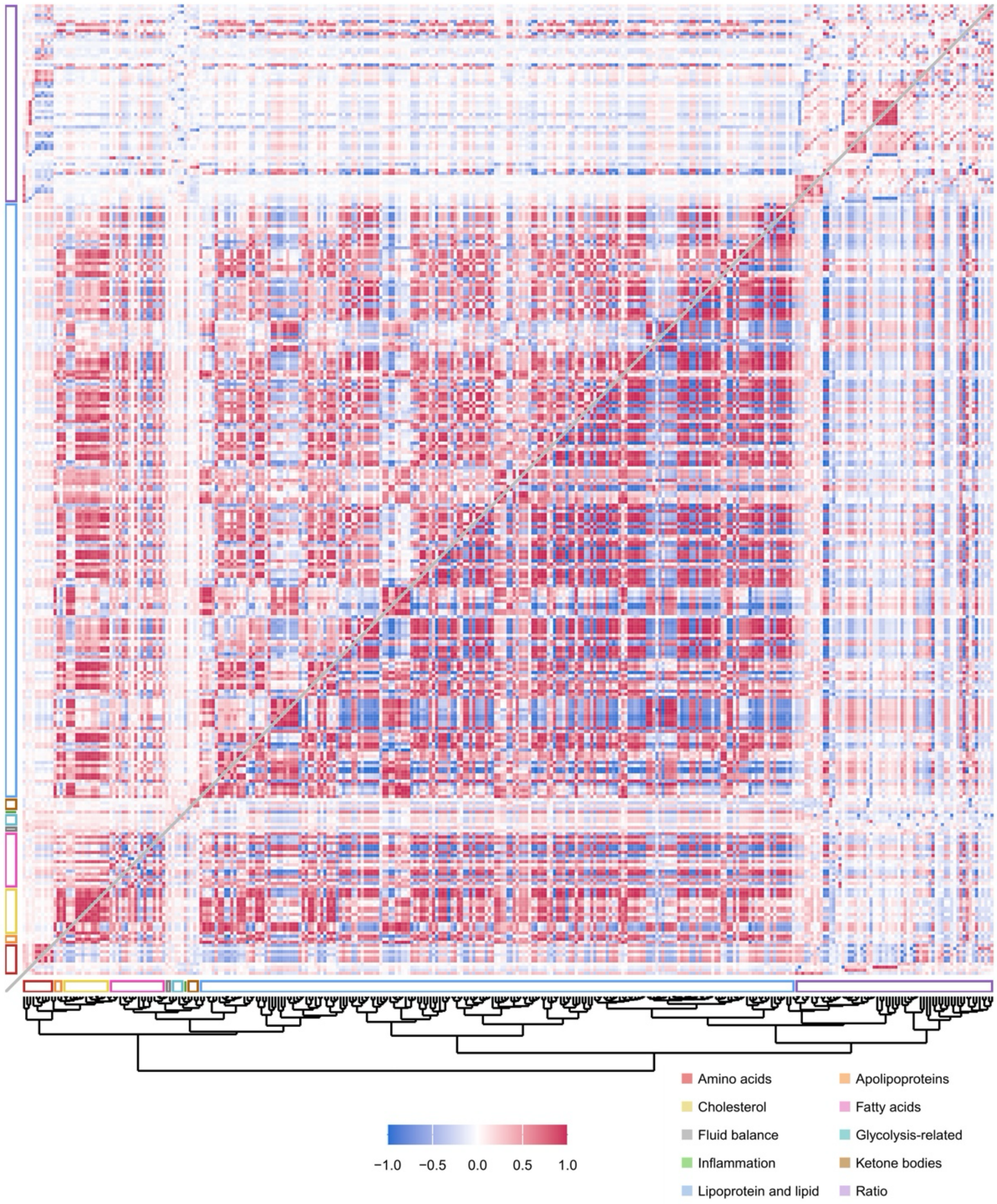

Supplementary Figure 3. Heat map illustrating the phenotypic (top left inset) and genetic (bottom right inset) correlation structure of the metabolic phenotypes.

The hierarchical clustering tree displays the results of hierarchical clustering based on the genetic correlation of the metabolic traits. The colors on the horizontal and vertical axes indicate the metabolite groups.

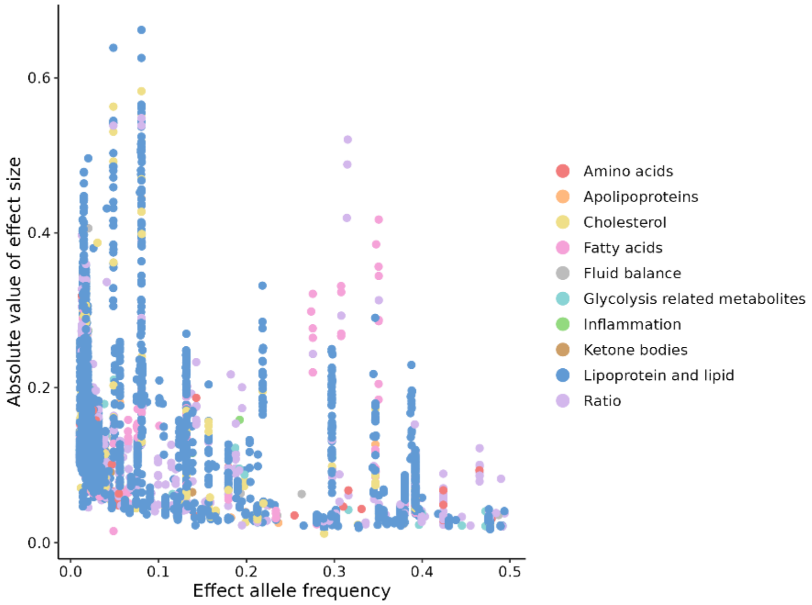

Supplementary Figure 4. Absolute effect size plotted against effect allele frequency for variants in 3,610 putative causal associations.

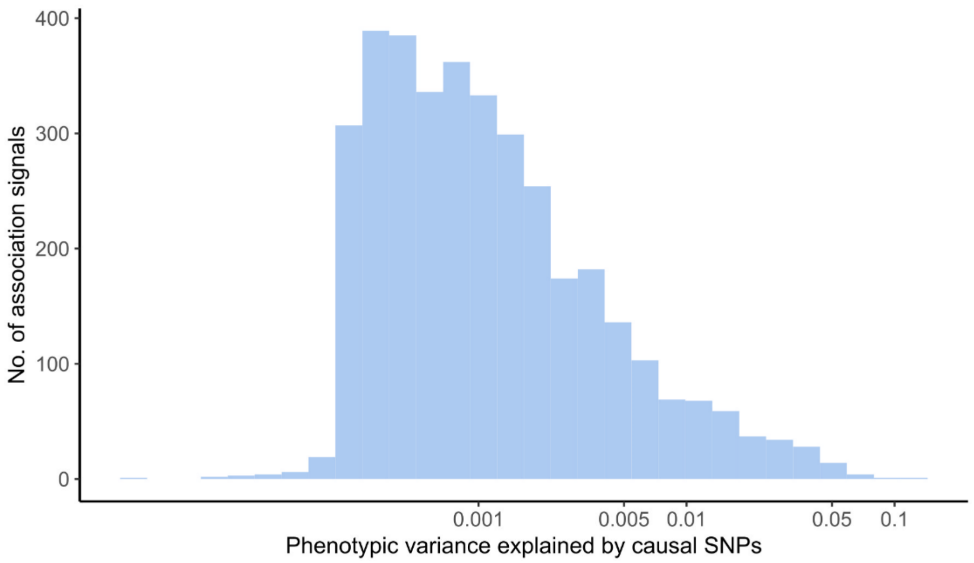

Supplementary Figure 5. Distribution of metabolite phenotypic variance explained by fine-mapped causal variants.

The distribution is plotted on the log scale.

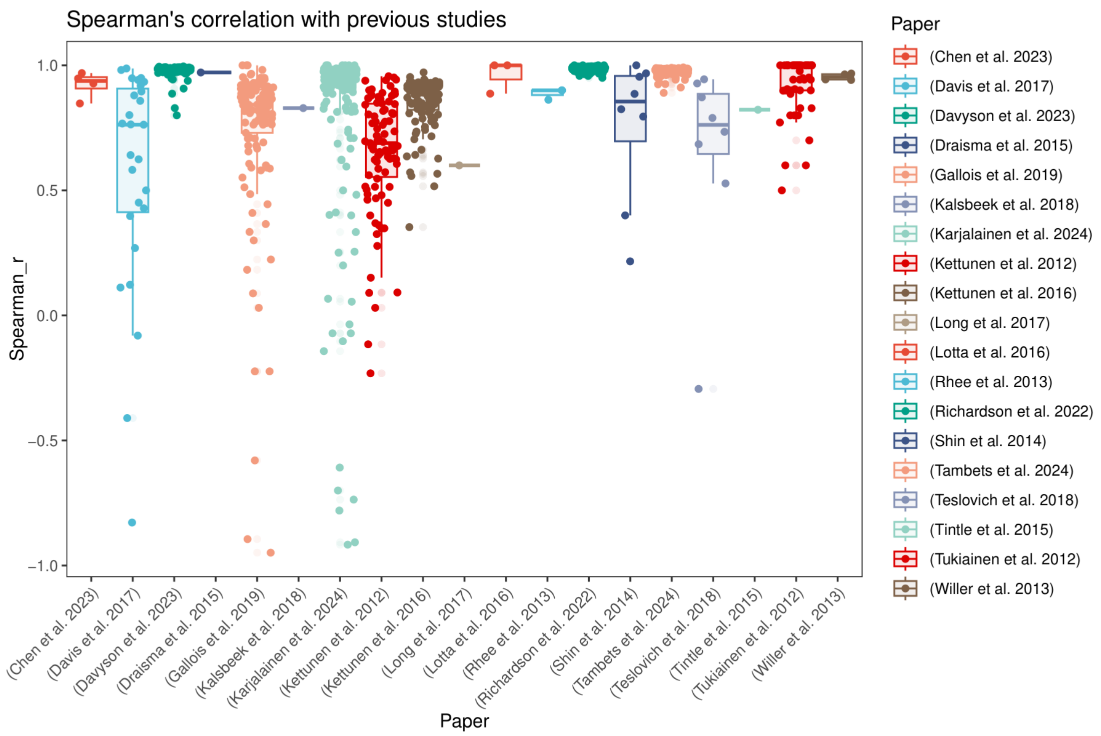

Supplementary Figure 6. Spearman's correlation of GWAS effect estimates across 19 studies

Spearman's correlation coefficients between the effect estimates in the current study and the effect estimates from 19 previous studies. Each point represents a correlation, with different colors indicating different papers. Data are presented as box plots, with the horizontal line indicating the median, boxes spanning the IQR, whiskers extending to the most extreme data point that is no more than 1.5 × IQR from the box edges, and points beyond representing outliers.

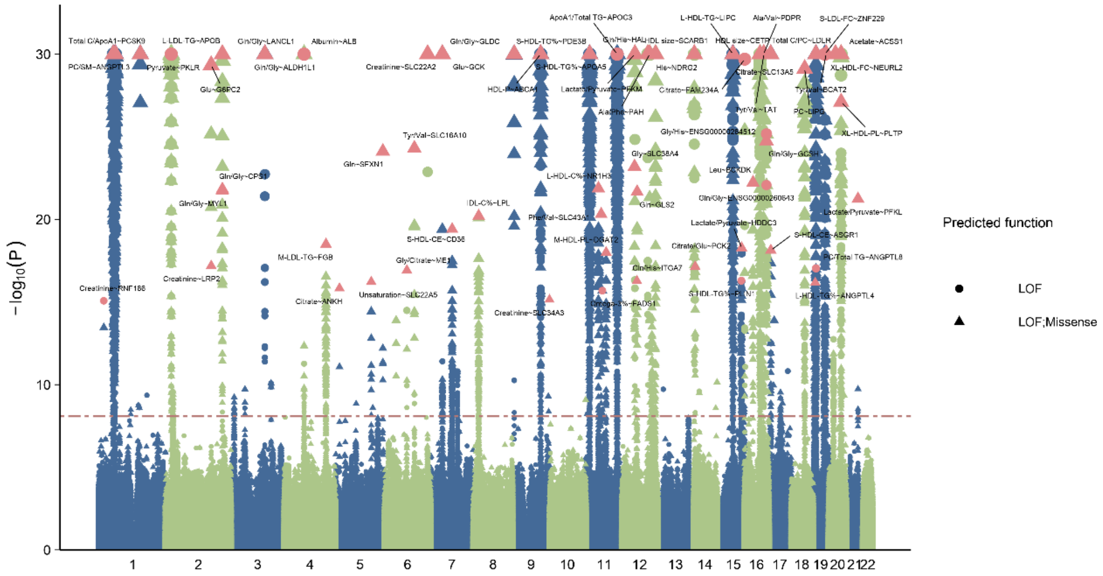

Supplementary Figure 7. Results of rare variant genomic aggregate testing

Manhattan plot displaying gene-level association results in 197,774 white British participants from the UKB. Point shapes represent different functional annotations. For each gene, the association with the lowest *P*-value is labeled and highlighted with a red point. The red dotted horizontal line denotes the significance threshold ($P = 8.17 \times 10^{-9}$). Chromosome boundaries are indicated by different colors. The $-\log_{10}(P)$ values are capped at 30 to improve the figure's readability.

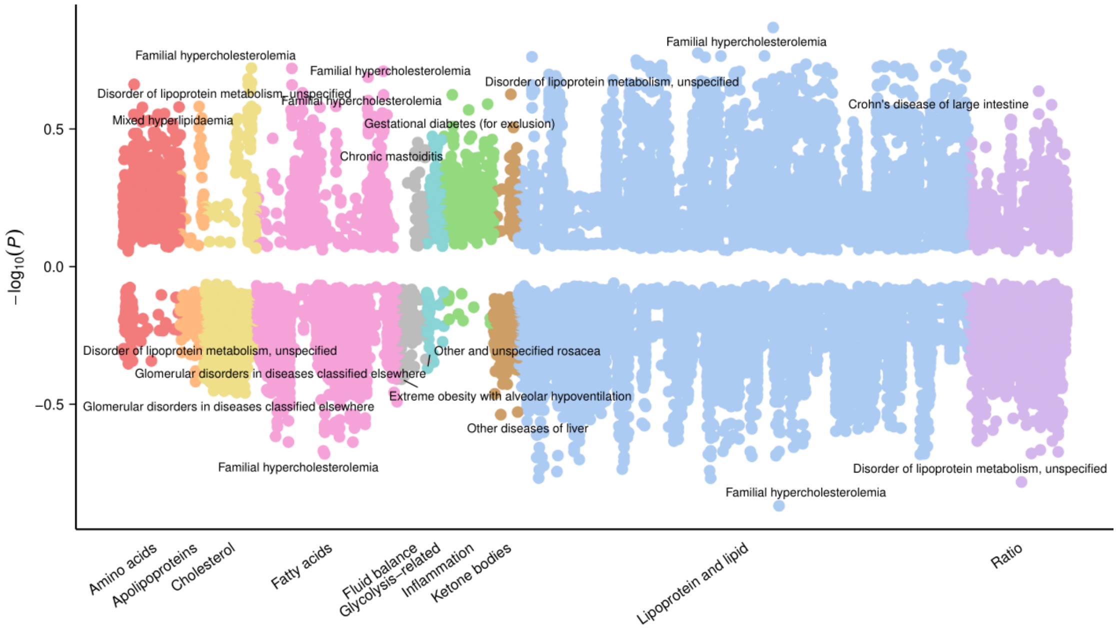

Supplementary Figure 8. Scatter plot displaying genetic correlations estimated for metabolic trait-disease pairs.

For each metabolite category, the diseases with the strongest positive or negative genetic correlations (above 0.3) are labeled. *P*-values are derived from genetic correlation analyses using LDSC. Only pairs with a $P < 0.01$ are included.

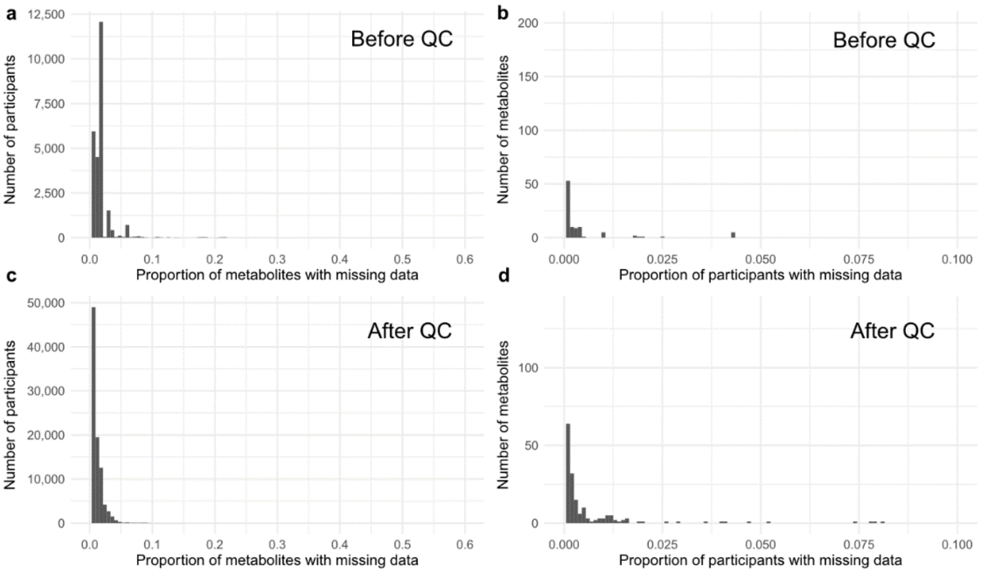

Supplementary Figure 9. Distribution of missing data across participants and metabolites before (a and b) and after (c and d) the quality control process.

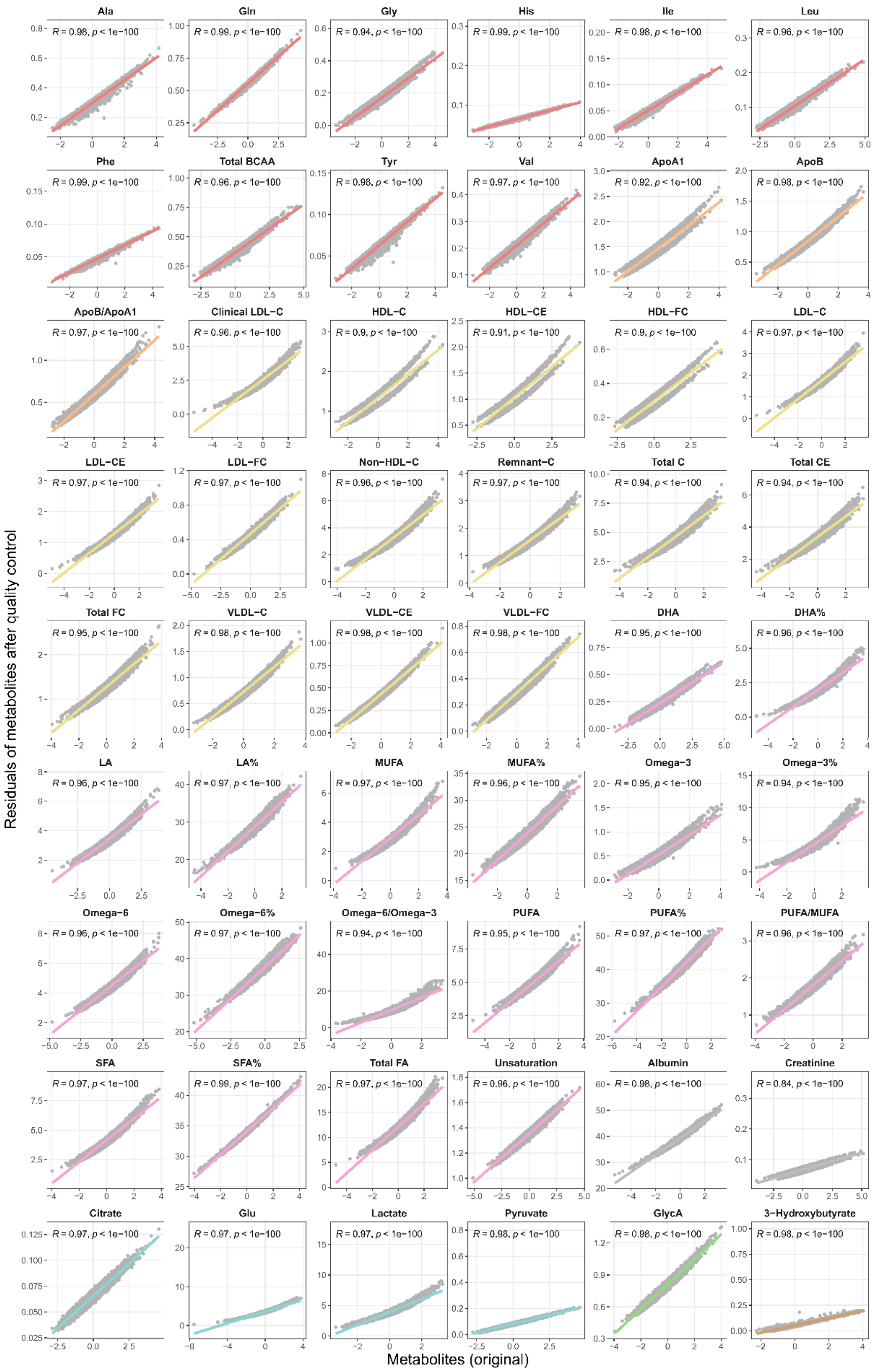
Ala
R = 0.98, p < 1e−100
Gln
R = 0.99, p < 1e−100
Gly
R = 0.94, p < 1e−100
His
R = 0.99, p < 1e−100
Ile
R = 0.98, p < 1e−100
Leu
R = 0.96, p < 1e−100
Phe
R = 0.99, p < 1e−100
Total BCAA
R = 0.96, p < 1e−100
Tyr
R = 0.98, p < 1e−100
Val
R = 0.97, p < 1e−100
ApoA1
R = 0.92, p < 1e−100
ApoB
R = 0.98, p < 1e−100
ApoB/ApoA1
R = 0.97, p < 1e−100
Clinical LDL−C
R = 0.96, p < 1e−100
HDL−C
R = 0.9, p < 1e−100
HDL−CE
R = 0.91, p < 1e−100
HDL−FC
R = 0.9, p < 1e−100
LDL−C
R = 0.97, p < 1e−100
LDL−CE
R = 0.97, p < 1e−100
LDL−FC
R = 0.97, p < 1e−100
Non−HDL−C
R = 0.96, p < 1e−100
Remnant−C
R = 0.97, p < 1e−100
Total C
R = 0.94, p < 1e−100
Total CE
R = 0.94, p < 1e−100
Total FC
R = 0.95, p < 1e−100
VLDL−C
R = 0.98, p < 1e−100
VLDL−CE
R = 0.98, p < 1e−100
VLDL−FC
R = 0.98, p < 1e−100
DHA
R = 0.95, p < 1e−100
DHA%
R = 0.96, p < 1e−100
LA
R = 0.96, p < 1e−100
LA%
R = 0.97, p < 1e−100
MUFA
R = 0.97, p < 1e−100
MUFA%
R = 0.96, p < 1e−100
Omega−3
R = 0.95, p < 1e−100
Omega−3%
R = 0.94, p < 1e−100
Omega−6
R = 0.96, p < 1e−100
Omega−6%
R = 0.97, p < 1e−100
Omega−6/Omega−3
R = 0.94, p < 1e−100
PUFA
R = 0.95, p < 1e−100
PUFA%
R = 0.97, p < 1e−100
PUFA/MUFA
R = 0.96, p < 1e−100
SFA
R = 0.97, p < 1e−100
SFA%
R = 0.99, p < 1e−100
Total FA
R = 0.97, p < 1e−100
Unsaturation
R = 0.96, p < 1e−100
Albumin
R = 0.98, p < 1e−100
Creatinine
R = 0.84, p < 1e−100
Citrate
R = 0.97, p < 1e−100
Glu
R = 0.97, p < 1e−100
Lactate
R = 0.97, p < 1e−100
Pyruvate
R = 0.98, p < 1e−100
GlycA
R = 0.98, p < 1e−100
3−Hydroxybutyrate
R = 0.98, p < 1e−100
Residuals of metabolites after quality control
Metabolites (original)

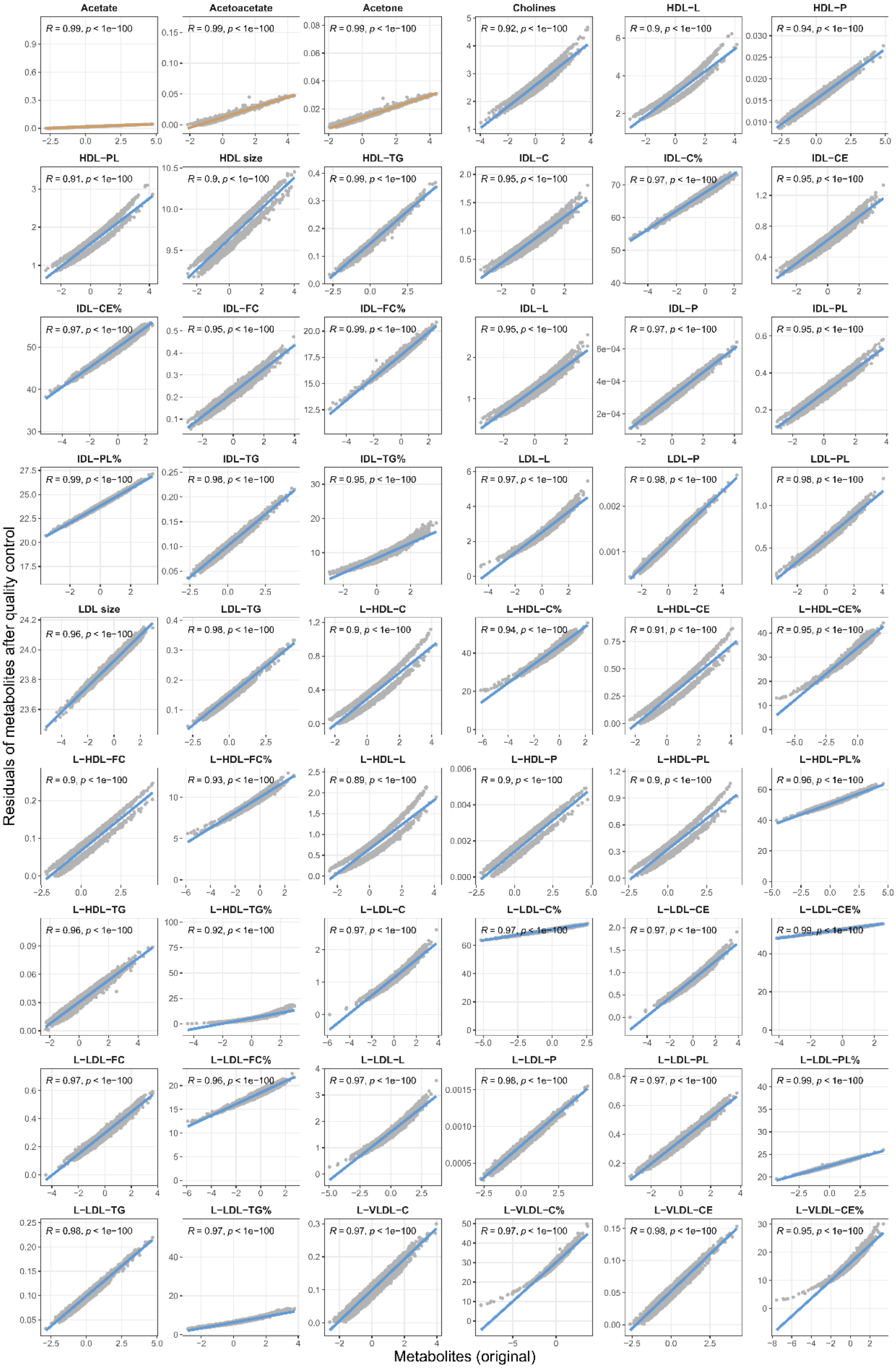
Residuals of metabolites after quality control
Metabolites (original)
Acetate
R = 0.99, p < 1e−100
Acetoacetate
R = 0.99, p < 1e−100
Acetone
R = 0.99, p < 1e−100
Cholines
R = 0.92, p < 1e−100
HDL−L
R = 0.9, p < 1e−100
HDL−P
R = 0.94, p < 1e−100
HDL−PL
R = 0.91, p < 1e−100
HDL size
R = 0.9, p < 1e−100
HDL−TG
R = 0.99, p < 1e−100
IDL−C
R = 0.95, p < 1e−100
IDL−C%
R = 0.97, p < 1e−100
IDL−CE
R = 0.95, p < 1e−100
IDL−CE%
R = 0.97, p < 1e−100
IDL−FC
R = 0.95, p < 1e−100
IDL−FC%
R = 0.99, p < 1e−100
IDL−L
R = 0.95, p < 1e−100
IDL−P
R = 0.97, p < 1e−100
IDL−PL
R = 0.95, p < 1e−100
IDL−PL%
R = 0.99, p < 1e−100
IDL−TG
R = 0.98, p < 1e−100
IDL−TG%
R = 0.95, p < 1e−100
LDL−L
R = 0.97, p < 1e−100
LDL−P
R = 0.98, p < 1e−100
LDL−PL
R = 0.98, p < 1e−100
LDL size
R = 0.96, p < 1e−100
LDL−TG
R = 0.98, p < 1e−100
L−HDL−C
R = 0.9, p < 1e−100
L−HDL−C%
R = 0.94, p < 1e−100
L−HDL−CE
R = 0.91, p < 1e−100
L−HDL−CE%
R = 0.95, p < 1e−100
L−HDL−FC
R = 0.9, p < 1e−100
L−HDL−FC%
R = 0.93, p < 1e−100
L−HDL−L
R = 0.89, p < 1e−100
L−HDL−P
R = 0.9, p < 1e−100
L−HDL−PL
R = 0.9, p < 1e−100
L−HDL−PL%
R = 0.96, p < 1e−100
L−HDL−TG
R = 0.96, p < 1e−100
L−HDL−TG%
R = 0.92, p < 1e−100
L−LDL−C
R = 0.97, p < 1e−100
L−LDL−C%
R = 0.97, p < 1e−100
L−LDL−CE
R = 0.97, p < 1e−100
L−LDL−CE%
R = 0.99, p < 1e−100
L−LDL−FC
R = 0.97, p < 1e−100
L−LDL−FC%
R = 0.96, p < 1e−100
L−LDL−L
R = 0.97, p < 1e−100
L−LDL−P
R = 0.98, p < 1e−100
L−LDL−PL
R = 0.97, p < 1e−100
L−LDL−PL%
R = 0.99, p < 1e−100
L−LDL−TG
R = 0.98, p < 1e−100
L−LDL−TG%
R = 0.97, p < 1e−100
L−VLDL−C
R = 0.97, p < 1e−100
L−VLDL−C%
R = 0.97, p < 1e−100
L−VLDL−CE
R = 0.98, p < 1e−100
L−VLDL−CE%
R = 0.95, p < 1e−100

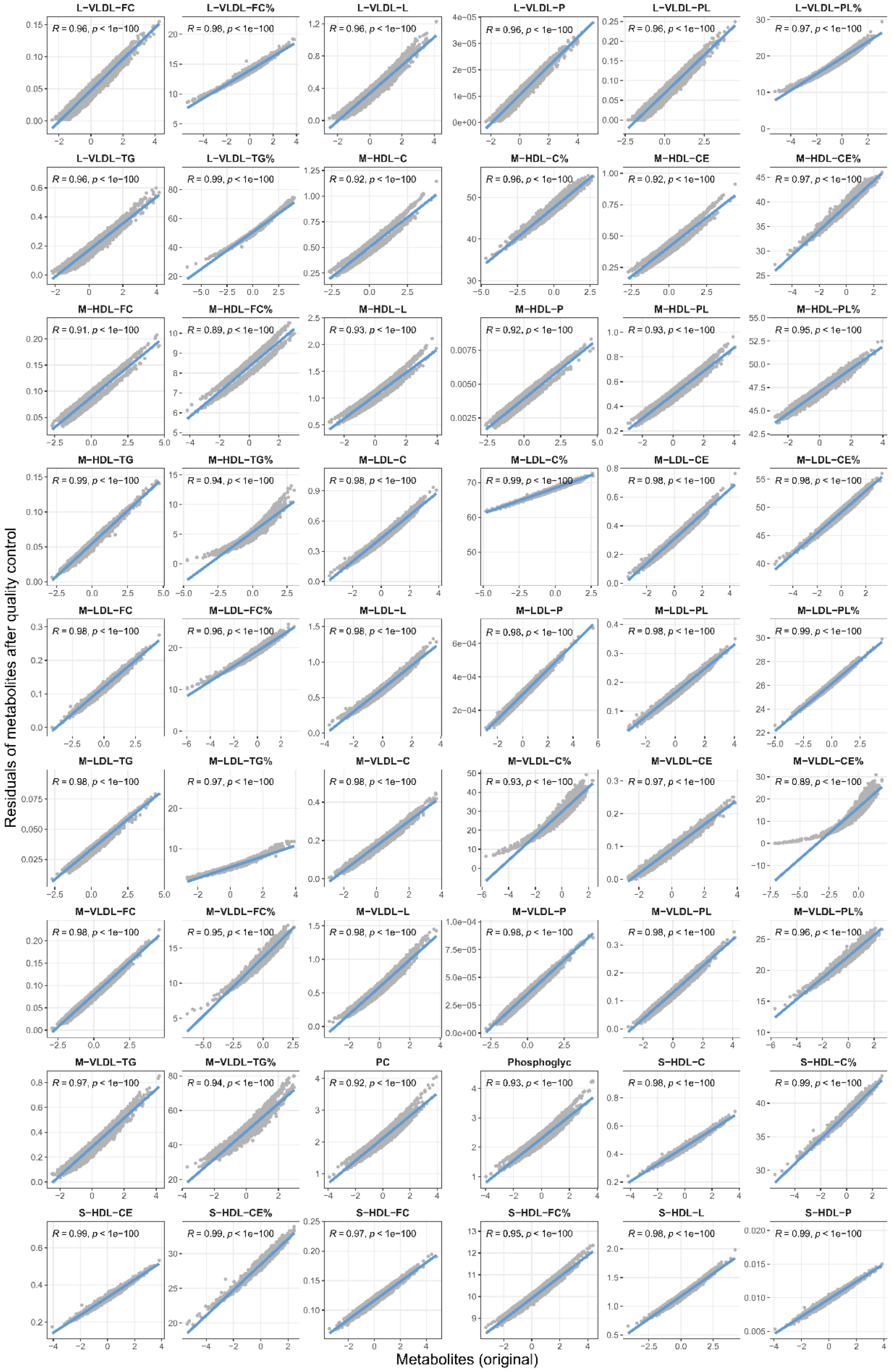
Residuals of metabolites after quality control
Metabolites (original)
L-VLDL-FC R = 0.96, p < 1e-100
L-VLDL-FC% R = 0.98, p < 1e-100
L-VLDL-L R = 0.96, p < 1e-100
L-VLDL-P R = 0.96, p < 1e-100
L-VLDL-PL R = 0.96, p < 1e-100
L-VLDL-PL% R = 0.97, p < 1e-100
L-VLDL-TG R = 0.96, p < 1e-100
L-VLDL-TG% R = 0.99, p < 1e-100
M-HDL-C R = 0.92, p < 1e-100
M-HDL-C% R = 0.96, p < 1e-100
M-HDL-CE R = 0.92, p < 1e-100
M-HDL-CE% R = 0.97, p < 1e-100
M-HDL-FC R = 0.91, p < 1e-100
M-HDL-FC% R = 0.89, p < 1e-100
M-HDL-L R = 0.93, p < 1e-100
M-HDL-P R = 0.92, p < 1e-100
M-HDL-PL R = 0.93, p < 1e-100
M-HDL-PL% R = 0.95, p < 1e-100
M-HDL-TG R = 0.99, p < 1e-100
M-HDL-TG% R = 0.94, p < 1e-100
M-LDL-C R = 0.98, p < 1e-100
M-LDL-C% R = 0.99, p < 1e-100
M-LDL-CE R = 0.98, p < 1e-100
M-LDL-CE% R = 0.98, p < 1e-100
M-LDL-FC R = 0.98, p < 1e-100
M-LDL-FC% R = 0.96, p < 1e-100
M-LDL-L R = 0.98, p < 1e-100
M-LDL-P R = 0.98, p < 1e-100
M-LDL-PL R = 0.98, p < 1e-100
M-LDL-PL% R = 0.99, p < 1e-100
M-LDL-TG R = 0.98, p < 1e-100
M-LDL-TG% R = 0.97, p < 1e-100
M-VLDL-C R = 0.98, p < 1e-100
M-VLDL-C% R = 0.93, p < 1e-100
M-VLDL-CE R = 0.97, p < 1e-100
M-VLDL-CE% R = 0.89, p < 1e-100
M-VLDL-FC R = 0.98, p < 1e-100
M-VLDL-FC% R = 0.95, p < 1e-100
M-VLDL-L R = 0.98, p < 1e-100
M-VLDL-P R = 0.98, p < 1e-100
M-VLDL-PL R = 0.98, p < 1e-100
M-VLDL-PL% R = 0.96, p < 1e-100
M-VLDL-TG R = 0.97, p < 1e-100
M-VLDL-TG% R = 0.94, p < 1e-100
PC R = 0.92, p < 1e-100
Phosphoglyc R = 0.93, p < 1e-100
S-HDL-C R = 0.98, p < 1e-100
S-HDL-C% R = 0.99, p < 1e-100
S-HDL-CE R = 0.99, p < 1e-100
S-HDL-CE% R = 0.99, p < 1e-100
S-HDL-FC R = 0.97, p < 1e-100
S-HDL-FC% R = 0.95, p < 1e-100
S-HDL-L R = 0.98, p < 1e-100
S-HDL-P R = 0.99, p < 1e-100

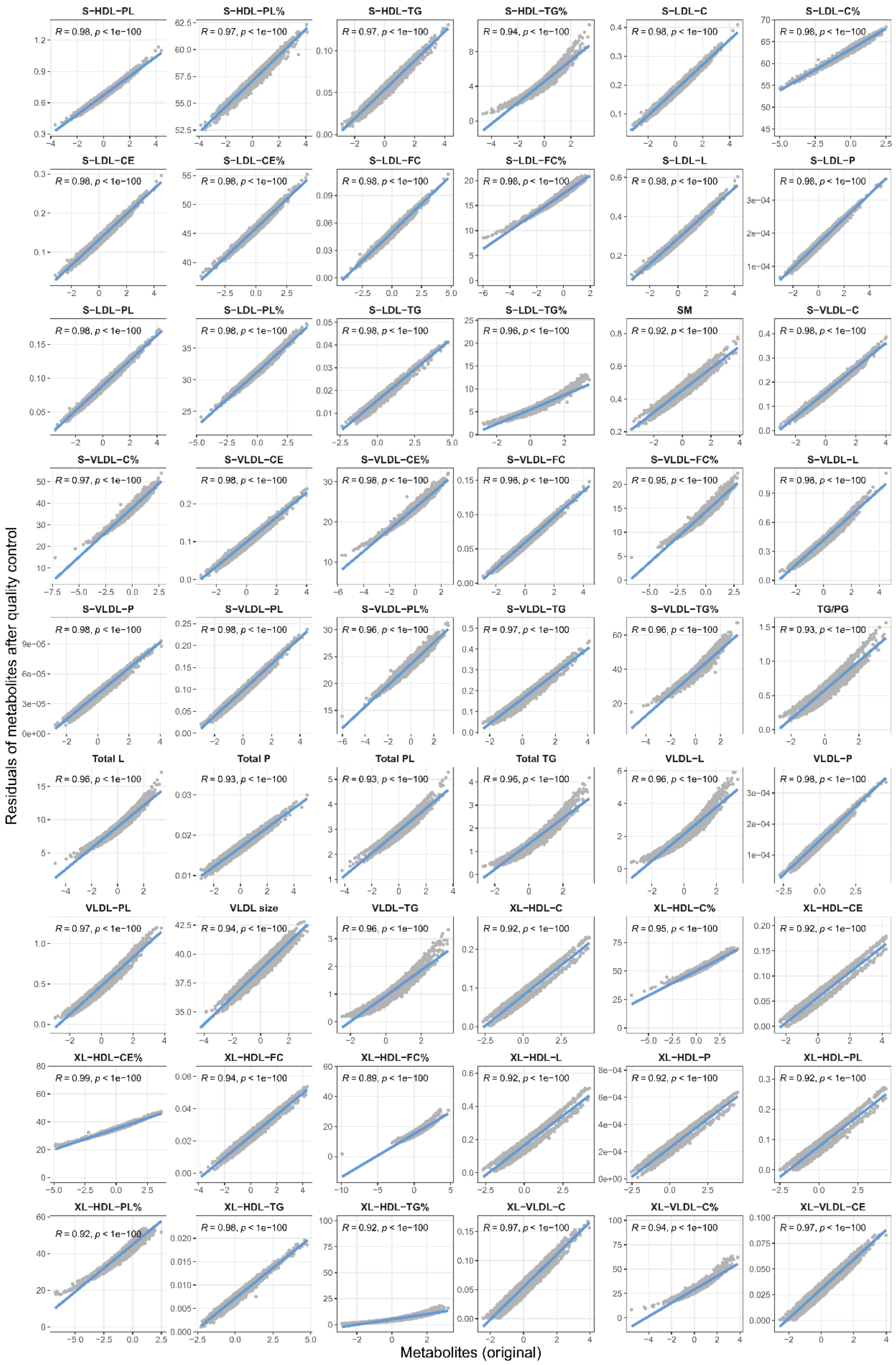
S-HDL-PL
R = 0.98, p < 1e-100
S-HDL-PL%
R = 0.97, p < 1e-100
S-HDL-TG
R = 0.97, p < 1e-100
S-HDL-TG%
R = 0.94, p < 1e-100
S-LDL-C
R = 0.98, p < 1e-100
S-LDL-C%
R = 0.98, p < 1e-100
S-LDL-CE
R = 0.98, p < 1e-100
S-LDL-CE%
R = 0.98, p < 1e-100
S-LDL-FC
R = 0.98, p < 1e-100
S-LDL-FC%
R = 0.98, p < 1e-100
S-LDL-L
R = 0.98, p < 1e-100
S-LDL-P
R = 0.98, p < 1e-100
S-LDL-PL
R = 0.98, p < 1e-100
S-LDL-PL%
R = 0.98, p < 1e-100
S-LDL-TG
R = 0.98, p < 1e-100
S-LDL-TG%
R = 0.98, p < 1e-100
SM
R = 0.92, p < 1e-100
S-VLDL-C
R = 0.98, p < 1e-100
S-VLDL-C%
R = 0.97, p < 1e-100
S-VLDL-CE
R = 0.98, p < 1e-100
S-VLDL-CE%
R = 0.98, p < 1e-100
S-VLDL-FC
R = 0.98, p < 1e-100
S-VLDL-FC%
R = 0.95, p < 1e-100
S-VLDL-L
R = 0.98, p < 1e-100
S-VLDL-P
R = 0.98, p < 1e-100
S-VLDL-PL
R = 0.98, p < 1e-100
S-VLDL-PL%
R = 0.96, p < 1e-100
S-VLDL-TG
R = 0.97, p < 1e-100
S-VLDL-TG%
R = 0.96, p < 1e-100
TG/PG
R = 0.93, p < 1e-100
Total L
R = 0.96, p < 1e-100
Total P
R = 0.93, p < 1e-100
Total PL
R = 0.93, p < 1e-100
Total TG
R = 0.96, p < 1e-100
VLDL-L
R = 0.96, p < 1e-100
VLDL-P
R = 0.98, p < 1e-100
VLDL-PL
R = 0.97, p < 1e-100
VLDL size
R = 0.94, p < 1e-100
VLDL-TG
R = 0.96, p < 1e-100
XL-HDL-C
R = 0.92, p < 1e-100
XL-HDL-C%
R = 0.95, p < 1e-100
XL-HDL-CE
R = 0.92, p < 1e-100
XL-HDL-CE%
R = 0.99, p < 1e-100
XL-HDL-FC
R = 0.94, p < 1e-100
XL-HDL-FC%
R = 0.89, p < 1e-100
XL-HDL-L
R = 0.92, p < 1e-100
XL-HDL-P
R = 0.92, p < 1e-100
XL-HDL-PL
R = 0.92, p < 1e-100
XL-HDL-PL%
R = 0.92, p < 1e-100
XL-HDL-TG
R = 0.98, p < 1e-100
XL-HDL-TG%
R = 0.92, p < 1e-100
XL-VLDL-C
R = 0.97, p < 1e-100
XL-VLDL-C%
R = 0.94, p < 1e-100
XL-VLDL-CE
R = 0.97, p < 1e-100
Residuals of metabolites after quality control
Metabolites (original)

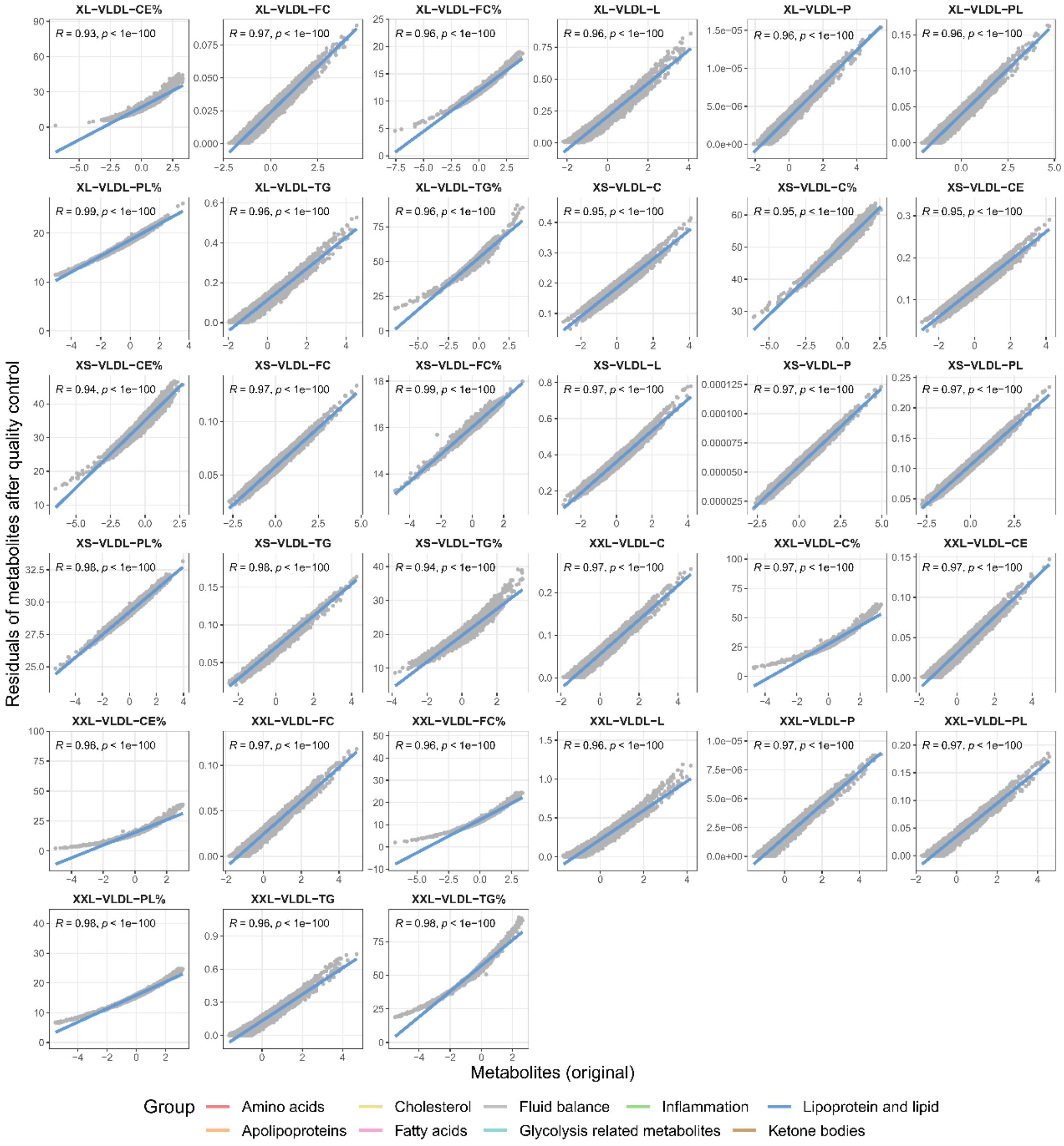

Supplementary Figure 10. Correlations between each of the raw and quality-controlled metabolites.

Pearson correlation coefficients and corresponding *P*-values are labeled.
