# Supplementary material for: Genetic architecture of plasma metabolome in 254,825 individuals
Source: Nat Commun. 2025 Sep 19;16:8272. doi: 10.1038/s41467-025-62126-w (PMC12449471; doi:10.1038/s41467-025-62126-w)
Supplement: Supplementary file 2 — Description of Additional Supplementary Files [file 41467_2025_62126_MOESM2_ESM.pdf]

Description of Additional Supplementary Files

Supplementary Data 1: Summary of participant characteristics

Supplementary Data 2: UKB Nightingale metabolite measurements and parameters

Supplementary Data 3: Summary of significantly associated SNPs and lead SNPs for each metabolite

Supplementary Data 4: Identified independent trait-associated loci associated with the 249 metabolic measures and 64 derived ratiosc

Supplementary Data 5: Summary of proteins shared by 64 metabolite pairs

Supplementary Data 6: Summary of numerator and denominator contributions to ratio-associated loci

Supplementary Data 7: Summary statistics for lead variants identified through ratio-based GWAS, including P values for constituent metabolites

Supplementary Data 8: Pleiotropic loci across the 249 metabolic measures and 64 derived ratios

Supplementary Data 9: Intercept values and estimated heritability from LD Score regression analysis

Supplementary Data 10: Novelty assessment and effector gene assignment for causal variants identified by fine-mapping

Supplementary Data 11: Number of associations with posterior probability greater than 0.95 or 0.99 and residual phenotypic variance explained by variants with > 99% posterior

inclusion probability

Supplementary Data 12: Genetic and phenotypic correlations between cholesterol measures in lipoprotein subclass particles

Supplementary Data 13: Information of 19 representative metabolomics GWAS used for validation

Supplementary Data 14: GWAS validation results across 19 studies

Supplementary Data 15: GWAS validation using variants filtered by multiple significance thresholds

Supplementary Data 16: Proportion of lead variants replicated between the current study and Karjalainen et al., 2024

Supplementary Data 17: GWAS validation results in individuals with non-British ancestries (Total N = 36,445)

Supplementary Data 18: Colocalization results supporting effector gene–metabolite annotations based on eQTL and pQTL data

Supplementary Data 19: The effector genes assigned to metabolites and metabolite ratios

Supplementary Data 20: Medical and pharmacological annotation of effector genes

Supplementary Data 21: Significant association signals from WES-based rare-variant aggregation tests and their significance after conditioning on nearby associated common lead variants identified in our GWAS

Supplementary Data 22: Genomic inflation factors of the WES-based aggregate test and the number of genes associated with each metabolite

Supplementary Data 23: Comparison of gene-metabolite associations between current study and Nag et al.

Supplementary Data 24: The FinnGen outcomes utilized in LDSC, colocalization and MR analyses

Supplementary Data 25: Significant colocalization results (PP.H4 > 0.8) between metabolic traits and FinnGen diseases

Supplementary Data 26: Significant MR results between metabolites and FinnGen diseases based on FDR-corrected IVW or wald ratio P-values (threshold 5e-8, IVs associated with >5 metabolites removed)

Supplementary Data 27: Subset of significant MR results (threshold 1e-6, IVs associated with >5 metabolites removed) based on pairs identified in the main MR analysis

Supplementary Data 28: Subset of significant MR results (threshold 5e-8, IVs associated with >3 metabolites removed) based on pairs identified in the main MR analysis

Supplementary Data 29: Significant reverse MR results based on FDR-corrected IVW or Wald ratio P-values, for pairs identified as significant in the main MR analysis

Supplementary Data 30: Sample sizes and UBERON ids for gene expression data across 49 tissues in GTEx V8

Supplementary Data 31: Instrumental variables associated with fewer than five metabolites (P < 5e-8)

Supplementary Data 32: Instrumental variables associated with fewer than five metabolites (P < 1e-6)
