# Supplementary material for: Genetic architecture of plasma metabolome in 254,825 individuals
Source: Nat Commun. 2025 Sep 19;16:8272. doi: 10.1038/s41467-025-62126-w (PMC12449471; doi:10.1038/s41467-025-62126-w)
Supplement: Supplementary file 4 — Reporting Summary [file 41467_2025_62126_MOESM4_ESM.pdf]

nature portfolio

Corresponding author(s): Jin-Tai Yu

Last updated by author(s): Jun 24, 2025

Reporting Summary

Nature Portfolio wishes to improve the reproducibility of the work that we publish. This form provides structure for consistency and transparency in reporting. For further information on Nature Portfolio policies, see our Editorial Policies and the Editorial Policy Checklist.

Statistics

For all statistical analyses, confirm that the following items are present in the figure legend, table legend, main text, or Methods section.

n/a	Confirmed	
☐	☒	The exact sample size (n) for each experimental group/condition, given as a discrete number and unit of measurement
☐	☒	A statement on whether measurements were taken from distinct samples or whether the same sample was measured repeatedly
☐	☒	The statistical test(s) used AND whether they are one- or two-sided<br>*Only common tests should be described solely by name; describe more complex techniques in the Methods section.*
☐	☒	A description of all covariates tested
☐	☒	A description of any assumptions or corrections, such as tests of normality and adjustment for multiple comparisons
☐	☒	A full description of the statistical parameters including central tendency (e.g. means) or other basic estimates (e.g. regression coefficient) AND variation (e.g. standard deviation) or associated estimates of uncertainty (e.g. confidence intervals)
☐	☒	For null hypothesis testing, the test statistic (e.g. F, t, r) with confidence intervals, effect sizes, degrees of freedom and P value noted<br>*Give P values as exact values whenever suitable.*
☐	☒	For Bayesian analysis, information on the choice of priors and Markov chain Monte Carlo settings
☒	☐	For hierarchical and complex designs, identification of the appropriate level for tests and full reporting of outcomes
☐	☒	Estimates of effect sizes (e.g. Cohen's d, Pearson's r), indicating how they were calculated

Our web collection on statistics for biologists contains articles on many of the points above.

Software and code

Policy information about availability of computer code

Data collection	No software is involved in data collection (all data used is directly available from the UK Biobank, as described in the manuscript)
Data analysis	See Methods section for the full details of the data analyses. The GWAS and clumping analyses were performed using PLINK (v2.0). Gene annotation was performed using bedtools (v2.31.0). LD score regression intercept, heritability, and genetic correlation effects were computed by LDSC (https://github.com/bulik/ldsc). Single-locus level pleiotropy and the polygenicity of metabolic traits were visualized using Fuji plot (https://github.com/yk-tanigawa/fujiplot) which is based on Circos (http://circos.ca). Fine-mapping was performed using FINEMAP (v1.4.21). All other data analyses were conducted using R (version 4.2.0). R packages used for analysis and plotting include dplyr (1.1.4), data.table (1.15.4), tidyverse (2.0.0), tidyr (1.3.1), stringr (1.5.1), CJAMP (0.1.1), rtracklayer (1.58.0), TwoSampleMR (0.6.1), MRInstruments (0.3.2), coloc (5.2.3), cowplot (1.1.3), RColorBrewer (1.1.3), ggplot2 (3.5.1), ggrepel (0.9.4), and ggraph (2.1.0). ANNOVAR, http://annovar.openbioinformatics.org/en/latest/; Locuszoom, http://locuszoom.sph.umich.edu/locuszoom.

For manuscripts utilizing custom algorithms or software that are central to the research but not yet described in published literature, software must be made available to editors and reviewers. We strongly encourage code deposition in a community repository (e.g. GitHub). See the Nature Portfolio guidelines for submitting code & software for further information.

Data

Policy information about availability of data

All manuscripts must include a data availability statement. This statement should provide the following information, where applicable:

- Accession codes, unique identifiers, or web links for publicly available datasets
- A description of any restrictions on data availability
- For clinical datasets or third party data, please ensure that the statement adheres to our policy

The full summary statistics data have been deposited in the Figshare database at https://doi.org/10.6084/m9.figshare.29390471.v1. Individual-level genetic and phenotypic data from the UKB are available at https://biobank.ndph.ox.ac.uk/ by application. FinnGen disease GWAS is available at https://www.finngen.fi/en/access_results. The NHGRI-EBI GWAS Catalog is accessible at https://www.ebi.ac.uk/gwas/. The PubMed database can be accessed via https://pubmed.ncbi.nlm.nih.gov/. The HMDB database can be found at https://hmdb.ca/. The KEGG pathway database can be found at https://www.genome.jp/kegg/pathway.html. The PubChem database can be found at https://pubchem.ncbi.nlm.nih.gov/. The GTEx V8 release data can be found at https://www.gtexportal.org/home/datasets. The UniProt database can be found at https://www.uniprot.org/uniprot/. The GENCODE resource can be found at https://www.gencodegenes.org/. The OMIM database can be found at https://www.omim.org/downloads. The IMPC database can be found at https://www.mousephenotype.org/data/release. The DrugBank database can be found at https://go.drugbank.com. Source data are provided with this paper.

Research involving human participants, their data, or biological material

Policy information about studies with human participants or human data. See also policy information about sex, gender (identity/presentation), and sexual orientation and race, ethnicity and racism.

Reporting on sex and gender	We took sex into consideration in our study and our analyses were adjusted for sex. Sex in the UKB was determined based on self-reporting data via questionnaires, and all included participants gave written informed consent for sharing of individual-level data. For significant associations identified in the primary analysis, we additionally performed sex-stratified sensitivity analyses.
Reporting on race, ethnicity, or other socially relevant groupings	Ethnic background information of the UKB participants was collected based on self-reported data via questionnaires.
Population characteristics	We included UKB participants of diverse ancestries, each with both metabolomic and genomic data available. The demographic profile of the study population revealed a median age of 58 years (interquartile range, IQR: 50-63 years), with 137,089 (53.79%) individuals being female and 218,380 (85.70%) individuals of white British ancestry. The median fasting time before blood collection was 3 hours (IQR: 2-4 hours).
Recruitment	UKB is a population-based prospective cohort consisting of more than 500,000 individuals aged 40 to 69 years at baseline. Participants were enlisted from 22 assessment centers across the UK between 2006 and 2010 and underwent extended monitoring. As UKB participants volunteered to join the study, self-selection bias may be present. Compared to the general population, UKB participants tend to be healthier. However, genetic associations are generally considered less susceptible to such biases, and the large sample size helps mitigate potential limitations.
Ethics oversight	Participants provided informed consent via electronic signatures. Ethics approval was granted by the North West Multi-Centre Research Ethics Committee.

Note that full information on the approval of the study protocol must also be provided in the manuscript.

Field-specific reporting

Please select the one below that is the best fit for your research. If you are not sure, read the appropriate sections before making your selection.

[x] Life sciences [ ] Behavioural & social sciences [ ] Ecological, evolutionary & environmental sciences

For a reference copy of the document with all sections, see nature.com/documents/nr-reporting-summary-flat.pdf

Life sciences study design

All studies must disclose on these points even when the disclosure is negative.

Sample size	No statistical methods were used to predetermine sample sizes. We excluded participants who were officially withdrawn by UKB or had more than 20% missing metabolite values. This resulted in a final sample of 254,825 individuals with both metabolomic and genomic data available. Given the large sample size, which exceeds those of most previously published GWAS of circulating metabolites, we consider the study to be sufficiently powered to detect meaningful genetic associations.
Data exclusions	We excluded participants who were officially withdrawn by UKB or had more than 20% missing metabolite values.
Replication	To validate our genetic findings, we employed three approaches. First, we compared our results with 19 previously published metabolomics GWAS studies and 1 WES studies, and observed strong concordance. Second, we compared our findings with the latest metabolomics GWAS, with 73% of their lead variants replicated in our study. Third, we conducted a validation analysis of the lead variants identified in the British discovery cohort using individuals of non-British ancestries, showing high effect size correlation (median R = 0.96). In addition, our

	substantially larger sample size enabled the discovery of numerous unreported associations.
Randomization	We used this observational dataset to test for genetic associations with plasma metabolic traits. Covariates including age, ethnicity, sex, fasting time, month of assessment, genotype measurement batch, the top 40 genotype PCs, age indicators by sex interactions, and ethnicity by sex interactions were adjusted.
Blinding	Blinding is not applicable to this study as this study is observational.

Reporting for specific materials, systems and methods

We require information from authors about some types of materials, experimental systems and methods used in many studies. Here, indicate whether each material, system or method listed is relevant to your study. If you are not sure if a list item applies to your research, read the appropriate section before selecting a response.

Materials & experimental systems

n/a	Involved in the study
☒	☐ Antibodies
☒	☐ Eukaryotic cell lines
☒	☐ Palaeontology and archaeology
☒	☐ Animals and other organisms
☒	☐ Clinical data
☒	☐ Dual use research of concern
☒	☐ Plants

Methods

n/a	Involved in the study
☒	☐ ChIP-seq
☒	☐ Flow cytometry
☒	☐ MRI-based neuroimaging

Plants

Seed stocks	*Report on the source of all seed stocks or other plant material used. If applicable, state the seed stock centre and catalogue number. If plant specimens were collected from the field, describe the collection location, date and sampling procedures.*
Novel plant genotypes	*Describe the methods by which all novel plant genotypes were produced. This includes those generated by transgenic approaches, gene editing, chemical/radiation-based mutagenesis and hybridization. For transgenic lines, describe the transformation method, the number of independent lines analyzed and the generation upon which experiments were performed. For gene-edited lines, describe the editor used, the endogenous sequence targeted for editing, the targeting guide RNA sequence (if applicable) and how the editor was applied.*
Authentication	*Describe any authentication procedures for each seed stock used or novel genotype generated. Describe any experiments used to assess the effect of a mutation and, where applicable, how potential secondary effects (e.g. second site T-DNA insertions, mosiacism, off-target gene editing) were examined.*
